# Corneal structure and endothelial morphological changes after uneventful phacoemulsification in type 2 diabetic and nondiabetic patients

**DOI:** 10.5935/0004-2749.20210071

**Published:** 2021

**Authors:** João Nuno Beato, João Esteves-Leandro, David Reis, Manuel Falcão, Vítor Rosas, Ângela Carneiro, Fernando Falcão Reis

**Affiliations:** 1 Department of Ophthalmology, São João Hospital, Porto, Portugal; 2 Department of Surgery and Physiology, Faculty of Medicine, University of Porto, Porto, Portugal; 3 Faculty of Medicine, University of Porto, Porto, Portugal

**Keywords:** Cataract, Phacoemulsification, Diabetes mellitus, type 2, Diabetic retinopathy, Endothelium, corneal, Corneal pachymetry, Corneal endothelial cell loss, Catarata, Facoemulsificação, Diabetes mellitus tipo 2, Retinopatia diabética, Epitélio posterior, Paquimetria corneana, Perda de células endoteliais da córnea

## Abstract

**Purpose:**

The aim of this study was to compare corneal structure and endothelial morphological changes after uneventful phacoemulsification cataract surgery between type 2 diabetic and nondiabetic patients and to determine the preoperative and intraoperative factors that may predict greater endothelial cell density loss.

**Methods:**

Forty-five diabetic pa tients (45 eyes) and 43 controls (43 eyes) with age-related cataract were enrolled in this prospective observational study. Corneal (thickness and volume) and anterior segment parameters were measured by Scheimpflug tomography; endothelial cell density and morphology (coefficient of variation of cell size, hexagonal cells) were recorded using noncontact specular microscopy. Patients were evaluated preoperatively and at one and six months after surgery. Univariate and multivariate linear regression analyses were performed to evaluate the relationship between demographic, clinical, ocular, and intraoperative parameters and postoperative endothelial cell density changes at six months.

**Results:**

Significant postoperative endothelial cell loss occurred one month after surgery in both groups (p<0.001), which remained stable until month 6; there were no differences between patients with and without diabetes mellitus at any time point. The mean postoperative central corneal thickness at one and six months did not change significantly from the mean preoperative value in either group (p>0.05). Multivariate linear regression analysis showed that older age (p=0.042) and higher cataract grades (p=0.001) were significantly associated with greater endothelial cell density reduction at six-month follow-up.

**Conclusion:**

This study showed that older age and denser cataracts might be associated with greater endothelial cell density reduction after cataract surgery. Other factors, such as diabetes mellitus and preoperative anterior segment parameters, did not influence postoperative changes in endothelial cell density.

## Abstract (pt)

**Objetivo:**

Comparar a estrutura da córnea e as alterações mor fológicas endoteliais após cirurgia de catarata por facoemulsificação sem intercorrências entre pacientes com diabetes mellitus tipo 2 e não diabéticos; e determinar quais fatores pré e intra-operatórios relacionados com a maior redução da densidade celular endotelial.

**Métodos:**

Quarenta e cinco diabéticos (45 olhos) e 43 (43 olhos) controlos com catarata relacionada à idade foram incluídos neste estudo observacional prospectivo. Os parâmetros da córnea (espessura e volume) e do segmento anterior foram medidos pela tomografia Scheimpflug; a densidade e morfologia celular endotelial (coeficiente de variação do tamanho das células, células hexagonais) foram registrados usando microscopia especular não contato. Os pacientes foram avaliados no pré-operatório, 1 e 6 meses após a cirurgia. Foi realizada uma análise de regressão linear uni e multivariada para avaliar a relação entre os parâmetros demográficos, clínicos, oculares e intra-operatórios com a redução da densidade celular endotelial aos 6 meses.

**Resultados:**

Nos dois grupos houve uma perda significativa de células endoteliais ao 1º mês pós-operatório (p<0,001), que permaneceu estável até ao 6º mês; sem diferenças estatisticas entre os grupos diabetes mellitus e não diabetes mellitus em qualquer avaliação. A espessura média da córnea no pós-operatório central aos 1 e 6 meses não mudou significativamente em relação ao valor médio pré-operatório nos dois grupos (p>0.05). A análise de regressão multivariada linear mostrou que a idade avançada (p=0.042) e os graus mais elevados de catarata (p=0.001) foram significativamente associados à maior redução densidade celular endotelial aos 6 meses de seguimento.

**Conclusão:**

Este estudo mostrou que a idade avançada e as cataratas mais densas podem predispor a uma maior redução densidade celular endotelial após a cirurgia de catarata. Outros fatores, como diabetes mellitus e parâmetros pré-operatórios do segmento anterior, não influenciaram significativamente as alterações pós-operatórias da densidade celular endotelial.

## INTRODUCTION

Advances in modern cataract surgery, such as phacoemulsification technique, viscoelastic materials, and new intraocular lenses, have allowed this surgery to be an effective method of cataract removal and visual rehabilitation^(1)^. Although the safety of phacoemulsification has been markedly validated in recent years, surgeons should be aware of possible risk factors of more complicated eyes. Among the most likely preoperative risk factors for greater endothelial cell loss are older age, shorter eyes with shallower anterior chambers, small pupil size, and higher lens nucleus density^(2-7)^.

Several studies have investigated the relationship between elevated plasma glucose concentrations in diabetes mellitus (DM) and corneal morphological and functional properties. Some studies have shown that diabetic subjects have lower endothelial cell density (ECD), which is associated with greater morphological changes^(8-10)^ and greater corneal thickness^(9,11)^, whereas other studies did not find any statistical differences^(12,13)^. Similarly, several studies have investigated the effect of cataract surgery aggression on endothelial cell count and the morphology of diabetic patients, but no definite conclusions were drawn^(7,12-19)^.

The purpose of this work was to evaluate the occurrence of corneal structural and endothelial morphological changes after uneventful phacoemulsification in patients with and without type 2 DM and to determine which preoperative and intraoperative factors might predict greater endothelial cell loss after surgery.

## METHODS

This was a prospective observational study conducted at the Department of Ophthalmology, Centro Hospitalar e Universitário São João, Porto, Portugal, between September 2015 and March 2016. The study protocol adhered to the tenets of the Declaration of Helsinki and received local Institutional Review Board approval. Each participant provided informed consent before they were included in the study.

### Study population

We invited patients with type 2 DM with different stages of diabetic retinopathy (DR) and controls, aged 50 years or older, to participate in this study. The diagnosis of type 2 DM was based on patient medical history, HbA1c level ≥6.5%, and/or current use of antidiabetic medication. Nondiabetic ageand sex-matched individuals were used as controls. All study participants were Caucasian.

We used the same exclusion criteria for both groups: prior eye surgery or trauma; any eye corneal, retinal, or optic nerve pathology except DR; mature cataracts (brown/white); Goldmann applanation tonometry (IOP-GAT) >25 mmHg; pseudoexfoliation syndrome; current treatment with any form of glucocorticoids; and endothelial cell count <1500 cells/m^2^. Diabetic patients were excluded from the analysis if they had severe nonproliferative diabetic retinopathy (NPDR), proliferative DR (PDR), or diabetic macular edema. We did not include any patients with intraoperative complications or use of adjunctive procedures^(20)^.

### Sample size calculation

According to the findings by Mathew et al.^(13)^, the standard deviation for ECD loss in the non-DM group was 13%. For a type I error of 0.05 and type II error of 0.20 (80% power), considering a mean difference of relative ECD change ≥8% to be significant between the two groups, we determined that the minimal required sample size was 42 subjects in each group.

### Study protocol

#### Preoperative assessment

All patients underwent preoperative evaluation within two weeks before cataract surgery, including general anamnesis and comprehensive ophthalmologic examination. The full protocol has been described elsewhere^(21)^.

We used the IOL Master^®^ 500 (Carl Zeiss Meditec, Jena, Germany; software version 7.7) for ocular biometry. Central corneal thickness (CCT) and volume, as well as anterior chamber morphometry (depth [ACD], volume [ACV], and angle [ACA]) were evaluated using the Pentacam^®^ HR (Oculus, Wetzlar, Germany; software version 1.20r87) in automatic release mode.

Corneal endothelial morphological properties were assessed using the noncontact specular microscope Topcon^®^ SP-3000P. At each visit, after confirming good alignment of the eye, a series of three good-quality photographs of the central cornea was taken from each eye using the automatic mode with a low flash intensity. The same ophthalmologist (J.B.), who was unaware of the patients’ diabetes status, independently analyzed all photographs using the computer-assisted cell density determination and morphometric analysis (ImageNet system, version 3.5.5). A semiautomated technique was used in which the automatic cell outlines of each photograph were reviewed and then corrected manually^(22)^. Central ECD (cells⁄mm^2^), the coefficient of variation (CV) of the cell area, and the percentage of hexagonal cells were recorded. For the analysis, we used the average of the three measurements for each eye.

The type of cataract (cortical, nuclear, posterior subcapsular) and nucleus opacity grade (1 [mild] to 4 [white/ brown] severity grading system) were classified after pupillary dilatation. The DR grade was assessed in all diabetic patients using seven standard Early Treatment Diabetic Retinopathy Study fundus photographs.

### Surgical technique

All cataract surgeries were performed under topical anesthesia by experienced surgeons (i.e., surgeons who have performed more than 250 surgeries). Subjects underwent a standard coaxial 2.75-mm clear cornea phacoemulsification technique (Model Infiniti; Alcon Laboratories, Inc., Fort Worth, TX, USA) using an in-the-bag one-piece acrylic posterior chamber intraocular lens (Acrysof^®^ SA60AT [Alcon Laboratories, Inc.] or Akreos^®^ Adapt lens [Bausch & Lomb, Inc., Rochester, NY, USA]) implantation. In all patients, we used Provisc^®^ (sodium hyaluronate 10%; Alcon Laboratories, Inc.) as the ophthalmic viscoelastic device.

The same postoperative medication was prescribed to all patients, which consisted of 1 mg/mL dexamethasone, 0.3 mg/mL flurbiprofen, and 5 mg/mL levofloxacin eye drops, five times daily for one week, followed by gradually tapering over three weeks.

### Postoperative assessment

All subjects were reexamined at one and six months postoperatively using a similar protocol to the baseline visit, with the exception of ocular biometry.

### Data and statistical analysis

Statistical analysis was performed using SPSS^®^ statistical software (version 21.0 for Mac OS; SPSS Inc., Chicago, IL., USA). Normality was assessed using distribution plots and Kolmogorov-Smirnov tests. All comparisons between the DM and non-DM groups, as well as between the préand postoperative periods, were performed with parametric or nonparametric tests, according to the normality of data. A χ^2^ test was performed for comparison of categorical variables. Univariate and multivariate linear regression analyses, using generalized linear models, were performed to identify the potential demographic (age, gender), clinical (duration of DM, HbA1c levels), ocular (preoperative axial length, CCT, ACD, ACV, ACA) and intraoperative (cataract grade, cumulative dissipated energy [CDE]) variables associated with postoperative changes in ECD. For all analyses, statistical significance was set at p<0.05.

## RESULTS

Forty-five diabetic patients and 43 nondiabetic controls were enrolled in the study. The demographic and clinical characteristics of the DM and non-DM groups were comparable, except that HbA1c levels were higher (p<0.001, Mann-Whitney test) and mean cataract grade was lower (p=0.021, Mann-Whitney test) in the DM group (Table 1). Moreover, in the DM group, a longer duration of DM was significantly associated with higher HbA1c levels (*p*=0.008, χ^2^ test).

**Table 1:** Demographic, clinical, and intraoperative characteristics of the study population

	DM group (n=45)	Non-DM group (n=43)	*P*
Age (y)	72.7 ± 5.7	70.5 ± 6.3	0.085^1^
Female (η)	28 (63%)	26 (60%)	0.866^3^
Right eyes (n)	22 (49%)	29 (67%)	0.078^3^
Axial length, mm			
Preoperatively	22.9 ± 0.7	23.0 ± 0.8	0.700^1^
Mean keratometry, D			
Preoperatively	44.2 ± 1.5	44.2 ± 1.6	0.972^1^
ACD, mm			
Preoperatively	2.6 ± 0.3	2.7 ± 0.4	0.080^1^
ACV, mm^3^			
Preoperatively	126 ± 33	139 ± 35	0.077^1^
HbA1 c levels (%)	6.8 ± 1.0	5.5 ± 0.4	<0.001* ^2^
Duration of diabetes (y)	9.1 ± 8.0	n/a	n/a
DR stage (n)			
No apparent DR	39 (87%)	n/a	n/a
Mild to moderate NPDR	6(13%)		
Oral antidiabetic agents (n)	43 (96%)	n/a	n/a
Insulin treatment (n)	7(16%)	n/a	n/a
Intraoperative data			
Cataract grade	1.6 ± 0.6	1.9 ± 0.6	0.021* ^2^
CDE	9.3 ± 7.1	9.3 ± 6.4	0.997^1^
1OL power	22.1 ± 1.6	22.2 ± 1.8	0.816^2^
Acrysof®/Akreos®	37/8	37/6	0.624^3^

Data were derived from independent-samples *t* test^1^, Mann-Whitney test^2^, and χ^2^ test^3^. Continuous variables are reported as mean ± standard deviation.

* *p<*0.05= statistical significance; ACA= anterior chamber angle; ACD= anterior chamber depth; ACV= anterior chamber volume; CDE= cumulative dissipated energy; DM= diabetes mellitus; DR= diabetic retinopathy; HbA1c= glycated hemoglobin; NPDR= nonproliferative DR= mm, millimeters; n/a= not applicable; y= years.

### Corneal thickness and volume comparisons

There were no statistically significant differences in CCT or corneal volume measurements between the DM and non-DM groups at any time point (Table 2).

**Table 2:** Preoperative and postoperative measurements in the DM and non-DM groups

	DM group (n=45)	Non-DM group (n=43)	*P*
CCT^a^ (µm)			
Preoperatively	559 ± 38	559 ± 29	0.981^1^
∆1 mo	562 ± 35	560 ± 29	0.718^1^
∆6 mo	554 ± 32	566 ± 31	0.074^1^
Corneal volume (mm^3^)			
Preoperatively	61.0 ± 4.2	60.2 ± 3.1	0.318^1^
∆1 mo	62.3 ± 4.0	61.6 ± 3.2	0.364^1^
∆6 mo	61.1 ± 3.9	61.5 ± 3.6	0.578^1^
ECD (cell/mm^2^)			
Preoperatively	2408 ± 362	2421 ± 304	0.850^1^
∆1 mo	2057 ± 529	1919 ± 507	0.217^1^
∆6 mo	2030 ± 527	1921 ± 470	0.307^1^
CV of cell size (%)			
Preoperatively	35.9 ± 6.0	35.0 ± 6.8	0.786^1^
∆1 mo	35.2 ± 6.8	33.8 ± 5.2	0.209^1^
∆6 mo	33.8 ± 5.2	34.0 ± 8.2	0.621^1^
Hexagonal cells (%)			
Preoperatively	56.2 ± 9.5	56.4 ± 10.5	0.890^1^
∆1 mo	52.1 ± 10.0	50.3 ± 9.8	0.667^1^
∆6 mo	54.5 ± 9.3	53.9 ± 10.0	0.658^1^

Data were derived from independent-samples *t* test^1^, Mann-Whitney test^2^, or χ^2^ test^3^. Continuous variables are reported as mean ± standard deviation.

* *p<*0.05, statistical significance.

a Central corneal thickness was measured by Pentacam at the corneal vertex. CV= coefficient of variation of cell area; ECD= endothelial cell density; mm= millimeters; mo= month; µm= micrometer; ∆= variation.

The mean postoperative CCT at one and six months did not change significantly from the mean preoperative level in both the DM and non-DM groups (paired t test; p>0.05).

The mean corneal volume at one month follow-up was significantly greater than the preoperative value in both groups (DM group, p=0.016 vs. non-DM group, p<0.001; paired t test). However, at six months postoperatively, no significant change from the preoperative value was observed in the DM group (p=0.777), whereas it remained significantly higher in the non-DM group (p=0.001).

### Endothelial morphology comparisons

#### ECD comparisons

The mean preoperative ECD was 2408 ± 362 cells⁄mm^2^ and 2421 ± 304 cells⁄mm^2^ in the DM and non-DM groups, respectively (p=0.850, independent-samples t test). The ECD was observed to be significantly lower than the preoperative value at both oneand six-month follow-up in both groups (p<0.001, paired t test). There were no between-group differences in postoperative ECD (Table 2). There was no statistically significant variation in ECD between the first and sixth month in either group (paired t test; p>0.05).

Table 3 displays the postoperative corneal changes in the subjects with DM. There were no statistically significant differences between subgroups of DM duration or HbA1c levels, except for CCT variation in the subgroup analysis of HbA1c levels.

**Table 3:** Subgroup analysis of diabetic patients at six-month follow-up

			Age (y)	DM Female (n) duration (y)		HbA1c (%)	CCT Preop (µm)	CCT ∆6 mo, µm (%)	ECD preop (cells)	ECD ∆6 mo, cells (%)	CV of cell area preop (%)	CV ∆6 mo, %	Hexag preop (%)	Hexag ∆6 mo, %	
Duration of diabetes (y)		<10 (n=27)	74 ± 5	16 5 ± 2 (59%)		6.5 ± 0.6	561 ± 43	-7.6 ± 25.1 (-1.2 ± 4.1%)	2424 ± 333	-412 ± 344 (-18 ± 15%)	36 ± 5.4	-2.1 ± 5.8	56 ± 8	-2.8 ± 10	
		≥10	71 ± 6	12 16 ± 7		7.3 ± 1.3	556 ± 30	-1.6 ± 24.8	2384 ± 410	-326 ± 451	35 ± 5.8	-0.7 ± 4.6	56 ± 7	-2.6 ± 10	
		(n=18)		(67%)				(-0.2 ± 4.4%)		(-14 ± 18%)					
		*p*	0.080^1^	0.616^3^ <0.001* ^2^		0.026* ^2^	0.666^1^	0.433^1^	0.725^1^	0.471^1^	0.646^1^	0.402^1^	0.938^1^	0.945^1^	
	HbA1c levels (%)	<7.0 (n=28)	73 ± 5	16 (57%)	7 ± 5	6.3 ± 0.5	556 ± 40	+1.4 ± 18.9 (+0.3 ± 3.3%)	2424 ± 384	-370 ± 348 (-16 ± 15%)	35 ± 6.3	-1.6 ± 5.8	56 ± 8	-1.7 ± 10	
		≥7.0	72 ± 7	12	13 ± 9	7.8 ± 1.0	565 ± 33	-16.1 ± 30.0	2382 ± 332	-390 ± 457	36 ± 4.1	-1.4 ± 4.6	57 ± 7	-4.3 ± 10	
		(n=17)		(71%)				(-2.6 ± 5.0%)		(-16 ± 18%)					
			0.487^1^	0.367^3^	0.002* ^2^	<0.001* ^2^	0.421^1^	0.021* ^1^	0.715^1^	0.869^1^	0.804^1^	0.911^1^	0.517^1^	0.395^1^	

Data were derived from independent-samples *t* test^1^, Mann-Whitney test^2^, or χ^2^ test^3^. Continuous variables are reported as mean ± standard deviation.

* *p<*0.05= statistical significance; ACA= anterior chamber angle; ACD= anterior chamber depth; ACV= anterior chamber volume; CCT= central corneal thickness; CV= coefficient of variation; ECD= endothelial cell density; Hexag= hexagonal cells; mo= month; preop= preoperative; ∆= variation.

#### CV of cell area comparisons

The mean preoperative CVs were 35.9 ± 6.0 and 35.0 ± 6.8 in the DM and non-DM groups, respectively (p=0.786). There were no statistically significant differences between groups in the CV at the oneand six-month visits (paired t test; p>0.05; Table 2).

In the DM group, the CV at the one-month visit was not statistically different from the preoperative value (p=0.744), but it was significantly reduced at six months (p=0.037, paired t test). In the non-DM group, the mean postoperative CV at one and six months did not change significantly from the mean preoperative value (p=0.249 and p=0.504, respectively; paired t test).

#### Comparison of percentage of hexagonal cells

The pean preoperative percentage of hexagonal cells was 56.2 ± 9.5% and 56.4 ± 10.5% in the DM and non-DM groups, respectively (p=0.890). At the oneand six-month visits, there were no statistically significant differences between groups in the percentage of hexagonal cells (paired t test; p>0.05; Table 2).

In both groups, the percentage of hexagonal cells was observed to be lower than the preoperative value at the one-month visit (DM group, p=0.031; non-DM group, p<0.001, paired t test) but not at the six-month visit (DM group, p=0.371; non-DM group, p=0.143, paired t test).

### Factors influencing postoperative change in ECD

Multivariate linear regression adjusting for age, gender, DM, axial length, preoperative ECD, and relevant anterior segment Scheimpflug parameters (ACD, ACV, ACA) showed that older age and denser cataracts were significantly associated with the reduction in ECD (Table 4). In the fixed model, the ECD was found to decrease significantly by an average of 13 cells⁄mm^2^ for each one-year increase in age, whereas grade 2 and 3 cataracts are expected to be associated with a significantly greater ECD loss, on average 286 and 459 cells⁄mm^2^, respectively, in comparison with cataract grade 1 (Table 4). There was no significant difference in ECD loss between grade 2 and 3 cataracts (p=0.164).

**Table 4:** Multivariate regression analysis of the relative effects of the baseline clinical and ocular characteristics on postoperative ECD change

Parameter	ECD ∆ (mmHg)	
	B (95% Cl)	P
Age (y)	-13.10 (-25.71 to -0.49)	0.042^*^
Gender (male)	+ 12.75 (-151.60 to +177.10)	0.879
DM absence	-54.46 (-200.24 to +91.32)	0.464
AL preop (mm)	-12.25 (-140.77 to +116.28)	0.852
ACD preop (mm)	-144.82 (-682.99 to +401.35)	0.611
ACV preop (mm^3^)	+ 3.74 (-1.82 to+9.30)	0.187
ACA preop (°)	-16.04 (-35.70 to +3.61)	0.110
ECD preop (cells+nm^2^)	-0.06 (-0.27 to+0.14)	0.544
Cataract		
Grade 1	-	-
Grade 2	-285.84 (-444.54 to-127.34)	<0.001^*^
Grade 3	-459.43 (-723.36 to-195.51)	0.001^*^

Data were derived from generalized linear models.

* *p<*0.05= statistical significance. ACA= anterior chamber angle; ACD= anterior chamber depth; ACV= anterior chamber volume; AL= axial length; DM= diabetes mellitus; ECD= endothelial cell density; mm= millimeters; y= years; µm= micrometer; ∆variation. The remaining variables (DM duration, HbA1c levels, CCT, CV, CDE) did not influence the model and were excluded.

## DISCUSSION

This study evaluated the corneal structural and endothelial outcomes after uncomplicated phacoemulsification cataract surgery in type 2 diabetic and nondiabetic patients. Although previous studies have reported endothelial cell loss and variations in corneal thickness after cataract surgery in diabetic subjects, many of those studies did not provide information on important preoperative and intraoperative parameters, such as nuclear density score and metabolic control of diabetes.

Results from this study showed a comparable preoperative structural and endothelial corneal morphology between patients with and without type 2 diabetes. This is in agreement with the findings of previous reports that did not observe any statistical differences^(12,13)^, but in contrast to other studies that did^(8-10)^. The inability to detect differences consistently could be explained by a wide individual variation in the corneal damage caused by DM^(23)^, the relatively small sample sizes, and a great heterogeneity in the duration and degree of metabolic control of DM^(8)^.

Hyperglycemia is responsible for several biochemical and ultrastructural corneal abnormalities, including the accumulation of advanced glycosylated end-products, which can lead to a defect of cell adhesion^(24)^; the increased expression of metalloproteinase, which can damage the basement membrane and limit cell migration^(17)^; and the inhibition of endothelial Na+/k+ ATPase activity, resulting in corneal edema and reduced transparency^(25)^. For these reasons, diabetic corneas are believed to be more vulnerable to stress and trauma caused by cataract surgery. In clinical practice, endothelial cell analysis provides important clinical information on corneal function and viability. Because corneal endothelial cells have a limited capacity for repair, it is extremely important to recognize the true effect of chronic glycemic dysregulation on this structure and the effects of surgical trauma.

In line with previous studies, there was a significant decrease in ECD at month 1, which remained stable until month 6 in both groups. No differences in endothelial cell losses were observed between groups, as occurred in the studies of Mathew el al.^(13)^, Misra et al.^(16)^ and Fernandez-Munoz et al.^(18)^ (Table 5). However, these results differ from the meta-analysis of Tang et al.^(17)^ which showed that DM patients had a significantly greater endothelial loss than non-DM patients from the first day to 3 months postoperatively. Few reasons might explain this, particularly the subject’s characteristics of each sample. For example, in our study the presence of a significantly higher lens nucleus density in the non-DM group might have played an important role. In addition, the inclusion of a well-controlled DM group with short disease duration (median: 7 years, range 1 to 35) could have masked the effect of diabetes on the endothelium. Indeed, the authors decided to exclude subjects with maculopathy and more advanced stages of DR because many of them required adjuvant intravitreal treatment simultaneously with phacoemulsification. By using the same protocol in both groups, the observed corneal changes were exclusively caused by the surgery and not due to other procedures.

**Table 5:** Review of studies comparing endothelial cell density (ECD) and central corneal thickness (CCT) changes after phacoemulsification in DM patients

Study (year)	No. of patients (eyes) Age (y)				Female (n)	FU (mo) DM(y)			HbA1c (%)	DR stage	CCT preop (µm)	CCT ∆, µm		ECD preop (cells)	ECD ∆ (%)	CV of cell area (%)		CV ∆ (%)	Hexag (%)	Hexag ∆ (%)
Morikubo et al.^(14)^ (2004)^a^	Cont. 93 (93) 69 ± 9 DM2 93 (93) 69 ± 9				n.r.	1 n.r.			n.r.	All DR stages	542 ± 33 544 ± 37	n.r. (+0.04%) n.r. (+1.6%)^*^		2722 ± 348 2728 ± 404	3.2 ± n.r 7.2 ± n.r^*^	31 ± 7 31 ± 7		No ∆ difference	59 ± 10 58 ± 13	No ∆ difference
Lee et al.^(19)^ (2005)		Cont.	30 (30)	49 ± 8	14 (47%)		6	n.a	n.r.	n.a	n.r.		n.r.	n.r.	No ∆ differences		n.r.	No ∆ difference	n.r.	Significant ∆
		DM	30 (30)	48 ± 10	13 (43%)			9 ± 5		NPDR										difference^*^
		DM	30 (30)	50 ± 9	15 (50%)			10 ± 6		PDR										
Hugod et al.^(12)^ (2011)		Cont.	30 (30)	76 ± 9	n.r.		3	n.r.	CBGT	29 No DR	530 ± 32		529 ± 34	2623 ± 335	1.4 ± n.r		31 ± 6	No ∆ difference	58 ± 7	No ∆
		DM2	30 (30)	75 ± 9					7.1 ± 1.4	1 NPDR	549 ± 44		548 ± 44	2651 ± 411	6.2 ± n.r ^*^		34 ± 5		57 ± 8	difference
Mathew et al.^(13)^ (2011)^c^		Cont.	163 (163)	60 ± 9	n.r.		3	n.r.	n.r.	n.r.^e^	503 ± 27		523 ± 33	1921 ± 322	16.6 ± 13.0		32 ± 58	No ∆ difference	n.r.	No ∆ difference
		DM2	153 (153)	61 ± 9							510 ± 31^*^		528 ± 30	2018 ± 349^*^	19.2 ± 11.3		34 ± 57			
Dhasmana et al.^(15)^ (2014)^b^		Cont. DM2	60 (60) 60 (60)	61 ± 6 60 ± 6	30 (50%) 29 (48%)		3	n.r.	n.r.	n.r.	499 ± 30 509 ± 26		515 ± 26 536 ± 28^*^	2480 ± 400 2246 ± 528^*^	8.1 ± n.r 14.2 ± n.r ^*^		20 ± 5 24 ± 5^*^	No ∆ difference	78 ± 6 67 ± 7^*^	Significant ∆ difference^*^
Misra et al.^(16)^ (2015)^d^		Cont. DM2	23 (23) 28 (28)	74 ± 7 71 ± 8	29 (57%)		6	n.a 5.7 ± n.r. 12 ± n.r 7.5 ± n.r.		All DR stages	507 ± 37 529 ± 35		No ∆ differences	2384 ± 438 No ∆ differences 2254 ± 426			n.r.	n.r.	n.r.	n.r.
Fernandez-Munoz et al.^(18)^ (2018)		Cont. DM2	21 (21) 21 (21)	67 ± 10	n.r.		3	n.r. n.r.		NPDR	560 ± 41 572 ± 48		556 ± 40 565 ± 47	2173 ± 436 1875 ± 443 2249 ± 409 1595 ± 403			40 ± 3 48 ± 4^*^	46 ± 8 55 ± 5^*^	46 ± n.r. 55 ± n.r.	46 ± n.r. 50 ± n.r.^*^

* = Statistically significant difference at *p<*0.05. CCT= central corneal thickness; DM= diabetes mellitus; DR= diabetic retinopathy; NPDR= nonproliferative diabetic retinopathy; PDR= proliferative diabetic retinopathy; FU= follow-up; n.r= not reported; y= years; mmHg= millimeters of mercury; ∆= variation.

a = CCT was evaluated by Orbscan;

b = CCT was evaluated by noncontact specular microscope;

c = CCT was evaluated by contact ultrasound;^d^= CCT was evaluated by anterior segment optical coherence tomography;^e^= Individuals with prior laser treatment were excluded.

The observed reduction in ECD was larger than that reported by previous studies with one or two surgeons^(1,12,15,19)^. There are two possible explanations for the discrepancy between the results. First, the design of the study, which included multiple surgeons performing cataract surgeries, could be pointed out as a weakness of this research. Nevertheless, it might also be considered one of the strengths, because the inclusion of the results from diffe rent cataract surgeons can provide information that may be generalizable to a larger group of patients. In fact, our results are comparable with those of Mathew et al.^(13)^, who also used multiple surgeons. Alternatively, the greater endothelial cell loss might be related to the ophthalmic viscoelastic device used. In a study by Storr-Paulsen et al.^(26)^, sodium hyaluronate 3% dispersive viscoelastic (Vitrax^®^, Advanced Medical Optics, Inc., Santa Ana, CA, USA) provided greater protection to the endothelium when compared with sodium hyaluronate 1% cohesive viscoelastic (Healon^®^, Advanced Medical Optics, Inc.) or methylcellulose dispersive viscoelastic (Celoftal^®^, Alcon Laboratories, Inc.)^(26)^. In the present study, we used a cohesive viscoelastic (Provisc^®^).

Morphological changes of the corneal endothelium have been suggested to better reflect the vulnerability of the corneal endothelium to intraocular surgery rather than central ECD^(12)^; however, there are no consistent data regarding postoperative changes in diabetic patients. In our study, we found a significant drop in the percentage of hexagonal cells in both diabetic and nondiabetic subjects at the first month, but by the sixth month after surgery, this was no longer significantly different. This pattern of variation is likely caused by rearrangement of endothelial cells and cellular edema that occur at an early stage after surgery but that progressively recover to preoperative status^(12,27)^.

Corneal thickness has been shown to increase in the first postoperative days, caused by corneal edema after cataract surgery, and subsequently return to preoperative values in the following weeks^(17,18)^. This study confirmed that there were no significant changes in CCT at one and six months postoperatively in either group. On the other hand, corneal volume was increased at one month in both groups, returning to preoperative levels in the DM group and remaining elevated in the non-DM group. The difference in behaviors of the two corneal parameters over time can likely be explained by the larger area of evaluation in corneal volume (diameter of 10 mm centered on the corneal apex) as compared with the CCT (performed at the corneal apex). The changes detected in the non-DM group at the sixt-month visit were too small to be considered clinically relevant in subjects who had an ECD within the normal range.

Like previous studies, our multivariate analysis confirmed that older age^(2)^ and higher cataract grade^(2,4-6)^ were significantly correlated with greater endothelial cell loss at six-month follow-up. However, the current study failed to demonstrate any significant relation with axial length^(3,7)^ or anterior segment parameters^(4,5)^. The evaluation of anterior segment parameters in studies assessing endothelial cell changes after cataract surgery provides valuable information, because cataract surgery takes place within that limited space. Similar to our results, Reuschel et al.^(28)^ did not detect any association between ECD loss and anterior segment parameters (also evaluated by Scheimpflug imaging). This might be explained by the fact that in both studies, the phacoemulsification technique was performed through a 2.75-mm corneal incision, which maintains the stability of the anterior chamber throughout the procedure and thus minimizes the turbulence of rapid fluid infusion and the movement of nuclear fragments^(6)^.

In addition, the authors investigated the impact of phaco parameters on postoperative changes in ECD. Our study did not find any significant relationship between the amount of CDE and ECD loss^(1,29)^, which can be attributed to the fact that CDE may not be the appropriate parameter for measuring ultrasound energy delivery. On the other hand, some studies have shown that phaco time^(6,16,30)^ and irrigation volumes^(2)^ were significantly asso ciated with postoperative reductions in ECD, but these were not evaluated in our work.

Diabetic subgroup analysis did not demonstrate any relationship between disease duration or HbA1c levels and postoperative corneal changes^(13)^. As discussed previously, the moderate sample size and characteristics of the DM subjects (i.e., glycemic status, disease duration, DR stage) might have limited the results of the analysis.

The present study excluded patients with more advanced stages of DR (NPDR with maculopathy and PDR), mature cataracts (brown/white), and complicated surgeries; therefore, we cannot draw any conclusions about those particular groups of patients. Another limitation of the present study is the use of the cataract grading system. The Lens Opacities Classification System III was not used in this study; instead, we employed a different method commonly used method in the clinical setting.

In conclusion, this study showed that older age and denser cataracts may predispose patients to greater endothelial cell loss after unventful cataract surgery. Other factors, such as DM, preoperative anterior segment pa rameters, or CDE were not found to influence endothelial postoperative changes. Additional studies with sufficient statistical power and well-defined diabetic and control groups might clarify the role of long-term poor metabolic control on postoperative corneal structural and endothelial changes.
